# C-953-08. The Hidden Economics of Burn Care: Machine Learning Exposes the Real Drivers of Cost

**DOI:** 10.1093/jbcr/irag033.132

**Published:** 2026-04-06

**Authors:** Kaitlyn Malek, Tony Zhao, Anjay Khandelwal

**Affiliations:** Northeast Ohio Medical University, Akron, OH; Northeast Ohio Medical University, Akron, OH; Akron Children's Hospital, Akron, OH

## Abstract

**Introduction:**

Burn care is uniquely complex, blending surgical intensity, prolonged recovery, and multidisciplinary coordination. Yet most published cost analyses rely on charges or reimbursements adjusted by cost-to-charge ratios—methods that distort the true financial picture and obscure what actually drives hospital expenditures. To move beyond these limitations, we applied modern machine learning techniques directly to institutional cost data. This approach, to our knowledge the first of its kind in burn care, provides an unbiased framework to connect clinical complexity with real hospital-incurred expenses.

**Methods:**

We retrospectively examined 465 consecutive inpatients admitted to a regional academic burn center over a two-year period. Cost outcomes included Total Cost, Fixed Direct Cost (FDC), and Variable Direct Cost (VDC). Decision trees were used to create branching, diagnostic-style cost algorithms, while random forest models quantified variable importance through declines in predictive accuracy.

**Results:**

Machine learning produced clinically intuitive, algorithm-like cost pathways:

Total Cost: Procedure frequency emerged as the dominant driver. Patients with ≤5 procedures and short LOS represented the lowest-cost group (median $19111). Those with >15 procedures and prolonged LOS accounted for the highest costs (median $651369). Intermediate strata were defined by burn size (TBSA).

FDC: Patterns again began with procedure frequency, escalating with LOS and TBSA.

VDC: Ventilator use was the root node. Non-ventilated patients consistently clustered at lower cost, while ventilated patients with long LOS generated the highest VDC (median $448566).

Random forest models validated these pathways. LOS ranked as the strongest predictor (18% decline in accuracy when excluded), followed by procedure frequency, TBSA, and ventilator days. Inhalation injury and burn etiology contributed minimally.

**Conclusions:**

Direct machine learning analysis of hospital-incurred costs identified a concise set of key drivers: LOS, procedure frequency, burn size, and ventilator use. Decision trees offered interpretable “cost algorithms,” while random forests confirmed the relative weight of each factor. This study moves beyond cost-to-charge assumptions, demonstrating a reproducible and transparent framework to illuminate the true financial underpinnings of complex burn care.

**Applicability of Research to Practice:**

As health systems worldwide face rising costs and a shift toward value-based care, machine learning provides a powerful tool to uncover cost drivers with clarity and precision. For burn centers, these insights can inform staffing, procedural efficiency, and resource allocation. More broadly, this methodology can be adapted across surgical and inpatient specialties, offering international relevance in efforts to improve efficiency, equity, and sustainability of care delivery.

**Funding for the study:**

N/A.

